# Specific inhibition of serine/arginine-rich protein kinase attenuates choroidal neovascularization

**Published:** 2013-03-05

**Authors:** Zhenyu Dong, Kousuke Noda, Atsuhiro Kanda, Junichi Fukuhara, Ryo Ando, Miyuki Murata, Wataru Saito, Masatoshi Hagiwara, Susumu Ishida

**Affiliations:** 1 Department of Ophthalmology, Hokkaido University Graduate School of Medicine, Sapporo, Japan; 2 Laboratory of Ocular Cell Biology and Visual Science, Hokkaido University Graduate School of Medicine, Sapporo, Japan; 3 Department of Anatomy and Developmental Biology, Kyoto University Graduate School of Medicine, Kyoto, Japan

## Abstract

**Purpose:**

To investigate the applicability of serine/arginine-rich protein kinase (SRPK)-specific inhibitor, SRPIN340, for attenuation of choroidal neovascularization (CNV) formation using a mouse model.

**Methods:**

Laser photocoagulation was performed to induce CNV in C57BL/6J mice, followed by intravitreal injection of SRPIN340 or vehicle. Seven days after the treatment, the CNV size was evaluated using a flatmount technique. Protein levels of vascular endothelial growth factor (VEGF) and inflammation-associated molecules, such as monocyte chemoattractant protein (MCP)-1 and intercellular adhesion molecule (ICAM)-1, in the retinal pigment epithelium-choroid complex were measured with enzyme-linked immunosorbent assay. Expression levels of total *Vegf*, exon 8a-containing *Vegf* isoforms, and *F4/80* (a specific marker for macrophage) were assessed using real-time PCR.

**Results:**

SRPIN340 inhibited CNV formation in a dose-dependent manner. Compared with the vehicle, SRPIN340 significantly decreased the protein levels of VEGF, MCP-1, ICAM-1, and consequently inhibited macrophage infiltration. Furthermore, SRPIN340 suppressed the gene expression levels of total *Vegf* and exon 8a-containing *Vegf* isoforms.

**Conclusions:**

SRPIN340, a specific inhibitor of SRPK, suppressed *Vegf* expression and attenuated CNV formation. Our data suggest the possibility that SRPIN340 is applicable for neovascular age-related macular degeneration as a novel chemical therapeutics.

## Introduction

Age-related macular degeneration (AMD) is the primary cause of visual loss in developed countries [1-3]. In particular, neovascular AMD, characterized by choroidal neovascularization (CNV), is responsible for most cases of severe vision loss due to AMD [4,5]. CNV formation occurs through multifactorial processes involving the complex interaction of metabolic, genetic, and environmental factors. In particular, recent basic [6, 7] and clinical [8-10] investigations have provided strong evidence that growth factors and cytokines, such as vascular endothelial growth factor (VEGF), play a pivotal role in the process of pathological angiogenesis.

The cytokine VEGF had long been considered a proangiogenic factor, but Bates and his colleagues identified antiangiogenic *VEGF* isoforms, *VEGFxxxb* (xxx denotes the number of amino acids), generated as a result of distal splice site selection in exon 8 (i.e., exon 8b) during alternative splicing of *VEGF* messenger RNA precursor (pre-mRNA) [11]. Pro- and antiangiogenic *VEGF* isoforms are generated from the same *VEGF* gene via alternative splicing of mRNA. The alternative splicing of *VEGF* pre-mRNA is regulated by splicing regulatory factors, including various serine/arginine-rich (SR) proteins [12]. Previous studies have shown that SR protein kinase (SRPK) activates SRSF1 (SF2/ASF) and SRSF5 (SRp40), both of which favor splicing site selection at exon 8a during the splicing of *VEGF* pre-mRNA, and consequently lead to upregulation of proangiogenic *VEGF* isoforms [13]. We have developed a specific inhibitor for SRPK, SRPIN340 [14], and inhibition of SRPK with SRPIN340 suppressed retinal angiogenesis by reducing the ratio of proangiogenic to total *VEGF* isoforms at the mRNA level [15]. In this study, we investigated the therapeutic effect of SRPIN340 on CNV using the laser photocoagulation model and found that SRPIN340 suppressed *Vegf* expression and attenuated CNV formation.

## Methods

### Animals and induction of choroidal neovascularization

Eight-week-old C57BL/6J male mice (CLEA Japan, Tokyo, Japan) were used for this study. All animal experiments were approved by the Hokkaido University Animal Use Committee and conducted in accordance with the Association for Research in Vision and Ophthalmology Statement for the Use of Animals in Ophthalmic and Vision Research. Anesthesia was induced by intraperitoneal injection of pentobarbital (0.05 mg/g bodyweight), and pupils were dilated with topical 5% phenylephrine hydrochloride and 5% tropicamide. After anesthesia induction and pupil dilation, four laser spots were placed around the optic disc (532 nm, 200 mW power, 0.1 s, 75 μm spot size, Novus Spectra; Lumineis, Tokyo, Japan) using a slit-lamp delivery system with a cover glass as a contact lens. Laser spots with vitreous, retinal, or subretinal hemorrhage were excluded from the analysis.

### Serine/arginine-rich protein kinase inhibitor

**Figure 1:**
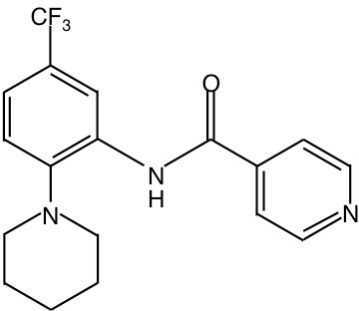
Structure of SRPIN340.

SRPIN340, an isonicotinamide compound, N-[2-(1-piperidinyl)-5-(trifluoromethyl)phenyl] (Figure 1), was found in a screening for chemicals specifically inhibiting SRPK to suppress the acute replication of viruses such as human immunodeficiency virus, using a scintillation proximity assay with a synthetic RS-repeat peptide as the substrate [16]. For SRPKs, SRPIN340 selectively inhibits SRPK1 and SRPK2 but does not inhibit other classes of SRPKs significantly, including Clk1 and Clk4 [14].

### SRPIN340 treatment

SRPIN340 (50 mM in 100% dimethyl sulfoxide, DMSO) was diluted with phosphate buffered saline (PBS, potassium chloride, 2.68 mM; potassium phosphate monobasic, 1.47 mM; sodium chloride, 136.89 mM; sodium phosphate dibasic, 8.10 mM) to various concentrations in 0.1% DMSO before treatment. Mice were divided into five groups: CNV induction alone (the control group) and CNV induction with 1 μl intravitreal injection of either 0.1% DMSO, 0.2 pmol, 2 pmol, or 20 pmol SRPIN340. Intravitreal injection was performed using a 33-gauge needle (Extreme Microsyringe, Ito Corporation, Tokyo, Japan) immediately after laser photocoagulation.

### Measurement of choroidal neovascularization

Seven days after laser injury, mice were euthanized with an overdose of anesthesia, performed by intraperitoneal injection with 2 mL of 5% pentobarbital sodium, and perfused with 5 mL PBS through the left ventricle, followed by 2.5 mL of 0.5% fluorescein-isothiocyanate-labeled dextran (Sigma-Aldrich, St. Louis, MO) in 1% gelatin. Subsequently, the eyes were enucleated and fixed with 2% paraformaldehyde for 30 min. Flat mounts of retinal pigment epithelium (RPE)-choroid complex were obtained by removing the anterior segments and the neural retina. Four to six radial relaxing incisions were made to allow the residual posterior eyecup to be laid flat. After mounting with the Vectashield Mounting Medium (Vector Laboratories, Burlingame, CA) and coverage with a coverslip, the flat mounts were examined with a fluorescence microscope (Biorevo, Keyence, Tokyo, Japan), and the CNV area was measured and used for the evaluation.

### RNA extraction and real-time polymerase chain reaction

Total RNA was extracted from the RPE-choroid complex 3 days after the laser photocoagulation procedure combined with intravitreal injection of either 1 μl of 0.1% DMSO or SRPIN340, following euthanasia by intraperitoneal injection of overdose pentobarbital (0.15 mg/g bodyweight) using TRIzol Reagent (Life Technologies, Carlsbad, CA). Reverse transcription was performed with GoScrip Reverse Transcriptase (Promega, Madison, WI) and oligo dT(20) primers following the manufacturer’s instructions. The primers used in this study are summarized in Table 1 [15]. The TaqMan probe for *F4/80* was purchased from Life Technologies. Real-time PCR was performed using the GoTaq qPCR Master Mix (Promega), THUNDERBIRD Probe qPCR Mix (TOYOBO, Tokyo, Japan), and StepOnePlus Real-Time PCR System (Life Technologies). *Actb* was used as endogenous control. Threshold cycle (C_T_) was determined automatically, and relative change in mRNA expression was calculated using the ΔΔCT values as previously reported [17]. All PCR reactions were repeated in triplicate, and the average values were used in the statistical analysis.

**Table 1:** Primer sequences.

Gene	Sequence (5’-3’)
*Vegf*	F: AAGGAGAGCAGA AGTCCCATGA
	R: CTCAATCGGACGGCAGTAGCT
*Vegf* containing exon 8a	F: GTTCAGAGCGGAGAAAGCAT
	R: TCACATCTGCAAGTACGTTCG
*Actb*	F: CATCCGTAAAGACCTCTATGCCAAC
	R: ATGGAGCCACCGATCCACA

*Vegf*, vascular endothelial growth factor; *Actb*, beta-actin

### Quantification of infiltrating macrophages

Immunostaining of F4/80 was also performed to further quantify the infiltration of macrophages. Briefly, eyes were enucleated 3 days after the laser photocoagulation in combined with 1 μl intravitreal injection of either 0.1% DMSO or 20 pmol SRPIN340, and whole choroid-sclera complexes were incubated overnight at 4 °C with a rabbit polyclonal antibody against mouse CD31 (1:100 dilution; Abcam, Tokyo, Japan) and a rat polyclonal antibody against F4/80 (1:100 dilution; Serotec, Oxford, UK) as primary antibodies, respectively. Binding of primary antibody was localized with Alexa Fluor 488 goat anti-rabbit and Alexa Fluor 546 goat anti-rat secondary antibody (1:200 dilution, respectively; Life Technologies). Finally, slides were mounted with Vectashield Mounting Medium (Vector Laboratories), and CNV was viewed with a fluorescence microscope (Biorevo, Keyence). The CD31-stained area of CNV and F4/80-positive macrophages were evaluated, and the area-adjusted number of macrophages per 1,000 μm^2^ area of CNV was calculated.

### Enzyme-linked immunosorbent assay

Four laser lesions were placed in each eye, and an intravitreal injection of either 1 μl of 0.1% DMSO or SRPIN340 was also administered. Protein levels of VEGF, monocyte chemoattractant protein (MCP)-1, and intercellular adhesion molecule (ICAM)-1 in supernatant were determined using enzyme-linked immunosorbent assay kits (R&D Systems, Minneapolis, MN) and normalized to total protein (BCA Protein Assay Kit, Thermo Scientific, Rockford, IL), according to the manufacturer’s protocols.

### Statistics

Results are presented as mean±standard error of the mean (SEM). Statistical analysis was performed using the two-tailed unpaired Student *t*-test, and results were considered statistically significant when the p value was less than 0.05.

## Results

### Impact of serine/arginine-rich protein kinase blockade during choroidal neovascularization formation

**Figure 2:**
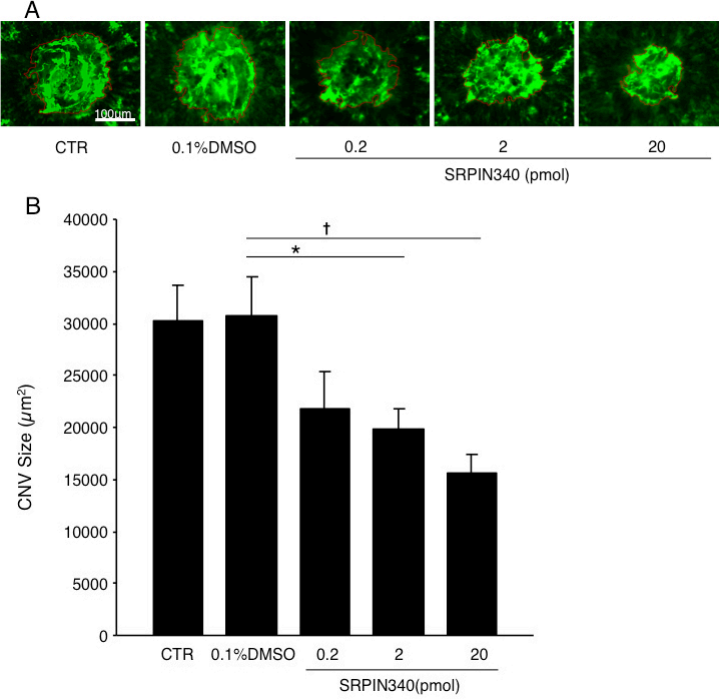
Suppression of choroidal neovascularization formation by SRPIN340. **A**: Representative micrographs of choroidal neovascularization (CNV) lesions in the choroidal flat mounts from mice treated with laser photocoagulation alone as control (CTR, n=32; n represents the number of CNV lesions), combined with intravitreal injection of 0.1% DMSO (n=31) or 0.2 pmol (n=17), 2 pmol (n=33), and 20 pmol (n=23) SRPIN340, respectively. **B**: Quantitative analysis of CNV size. Bars indicate the average of CNV size in each group. Values are mean±SEM. *, p<0.05; †, p<0.01.

To determine whether SRPK blockade inhibits CNV formation, we quantified the CNV size in the flat mounts of the RPE-choroid complex with or without SRPIN340 administration. Seven days after laser injury, the animals treated with 2 pmol SRPIN340 (n=33; n represents the number of CNV lesions) showed a significant decrease in the average CNV size (19,870±1935 μm^2^), compared with the vehicle-treated animals (30,737±3758 μm^2^, n=31, p<0.05; Figure 2A,B). Furthermore, a higher dose administration of SRPIN340 (20 pmol; n=23) significantly reduced the CNV size (15,649±1803 μm^2^, p<0.01) to an even greater extent than that observed in the 2 pmol SRPIN340–treated animals, whereas a lower dose administration (0.2 pmol; n=17) did not significantly inhibit CNV formation (21,741±3695 μm^2^, p=0.10; Figure 2A,B). No significant difference in CNV size was observed between mice subjected to laser injury alone and those subjected to laser and intravitreal injection of vehicle solution. The data indicate that SRPK blockade suppresses CNV growth in a dose-dependent manner.

### Impact of serine/arginine-rich protein kinase blockade on *Vegf* expression

**Figure 3:**
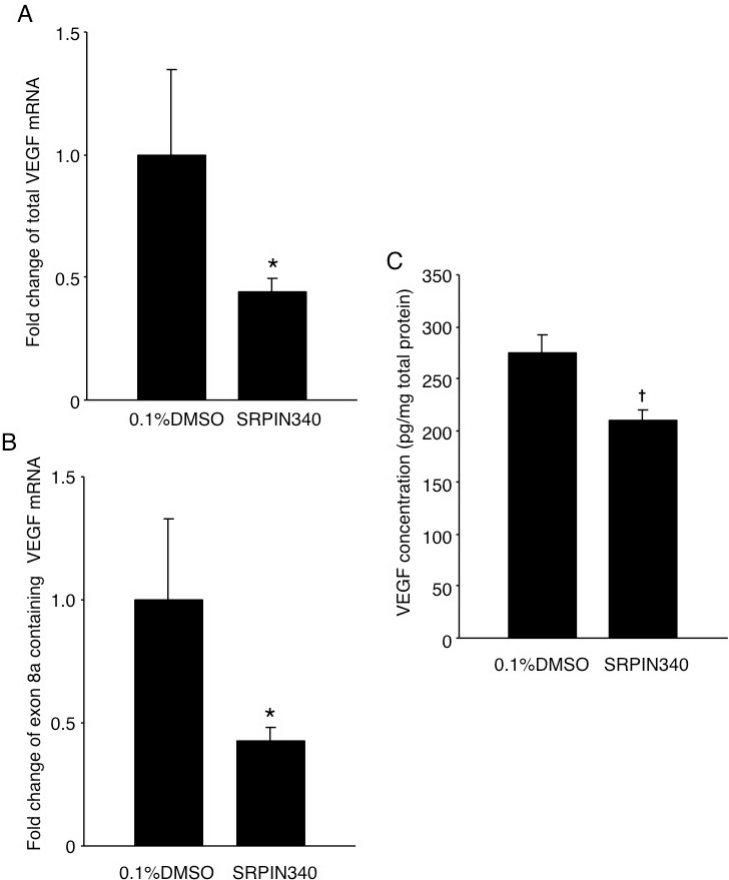
Inhibition of *Vegf* by SRPIN340. **A**: Bars represent real-time polymerase chain reaction (PCR) analysis of total *Vegf* mRNA. **B**: Bars represent real-time PCR analysis of exon 8a containing *Vegf* mRNA. **C**: ELISA of total VEGF protein in the RPE-choroid complex obtained from CNV mice 3 days after laser photocoagulation with intravitreal injection of 0.1% DMSO or 20 pmol SRPIN340 (n=6 to 8 for real-time PCR analysis, n=8 to 10 for ELISA, respectively; n represents the number of eyes). *, p<0.05; †, p<0.01.

To investigate the effect of SRPIN340 on *Vegf* isoforms, mRNA expression of total *Vegf* and *Vegf* isoforms containing exon 8a were analyzed using real-time PCR. Compared with mice treated with 0.1% DMSO (n=8; n represents the number of eyes), mRNA expression of total *Vegf* in the RPE-choroid complex obtained from mice treated with 20 pmol SRPIN340 (n=6) was significantly decreased by 56% (p<0.05; Figure 3A). Similarly, mRNA expression of *Vegf* containing exon 8a was significantly decreased by 57% (p<0.05; Figure 3B). Furthermore, total VEGF concentration in mice treated with 20 pmol SRPIN340 (209.2±10.9 pg/mg, n=8) was significantly lower than mice treated with 0.1% DMSO (274.2±17.9 pg/mg, n=10, p<0.01; Figure 3C).

### Suppression of adhesion molecules, inflammatory molecules, and macrophage influx by serine/arginine-rich protein kinase blockade

**Figure 4:**
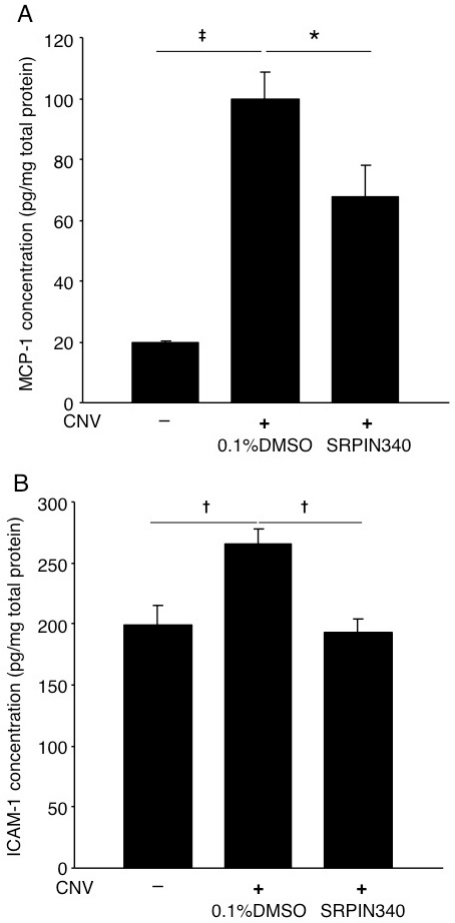
Reduction of inflammatory molecules by SRPIN340. **A, B**: Bars indicate average protein levels of monocyte chemoattractant protein (MCP)-1 and intercellular adhesion molecule (ICAM)-1 in the RPE-choroid complex obtained normal mice or CNV mice 3 days after laser photocoagulation combined with intravitreal injection of 0.1% DMSO or 20 pmol SRPIN340. Protein levels were measured with ELISA and normalized to total protein levels. Values are mean±SEM (n=8 to 10; n represents the number of eyes). *, p<0.05; †, p<0.01; ‡, p<0.001.

To explore the mechanism by which SRPIN340 suppresses CNV formation, concentrations of the inflammation-associated molecules MCP-1 and ICAM-1 in the RPE-choroid complex were measured. Levels of MCP-1 (19.5±0.9 pg/mg) and ICAM-1 (199.6±15.5 ng/mg) in the RPE-choroid complex of normal mice were significantly increased 3 days after laser photocoagulation and intravitreal injection of 0.1% DMSO (MCP-1, 99.9±8.6 pg/mg, p<0.001; ICAM-1, 265.4±12.3 ng/mg, p<0.01, respectively). However, compared with mice treated with laser photocoagulation and 0.1% DMSO, the protein levels of MCP-1 and ICAM-1 were significantly reduced in the RPE-choroid complex of the mice treated with laser photocoagulation and 20 pmol SRPIN340 (MCP-1, 67.8±10.2 pg/mg, p<0.05; ICAM-1, 192.9±11.6 ng/mg, p<0.01, n=8-10, respectively; n represents the number of eyes, Figure 4A,B).

**Figure 5:**
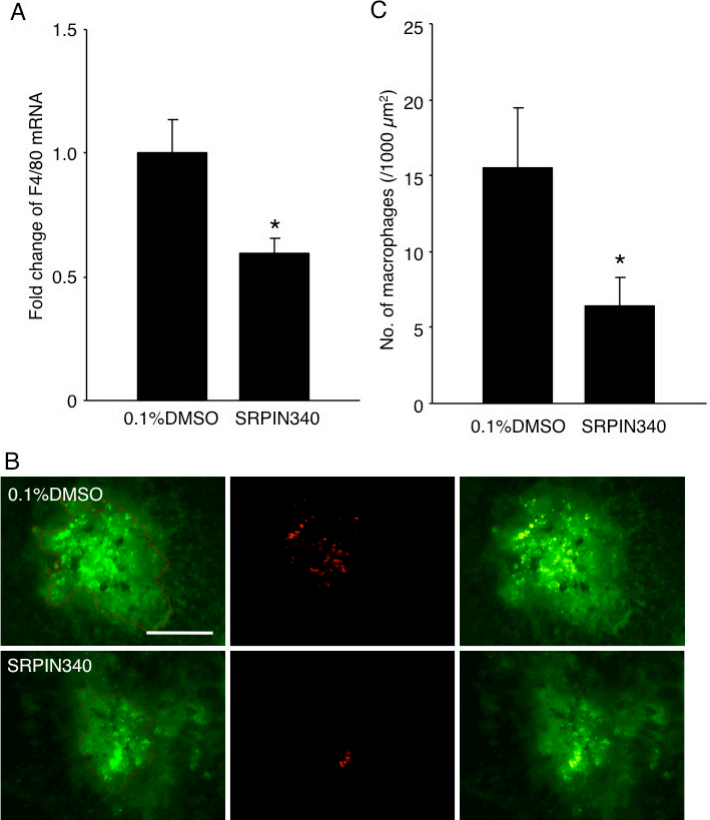
Inhibition of macrophage infiltration by SRPIN340. **A**: Bars represent real-time polymerase chain reaction (PCR) analysis of the relative change in *F4/80* expression in the RPE-choroid complex obtained from CNV mice 3 days after laser photocoagulation combined with intravitreal injection of 0.1% DMSO or 20 pmol SRPIN340. Values are mean±SEM (n=6 to 8; n represents the number of eyes). **B**: Micrographs depict representative *F4/80* immunostaining in CNV lesions 3 days after laser photocoagulation combined with intravitreal injection of 0.1% DMSO or 20 pmol SRPIN340. (left). CNV lesions stained for CD31 (middle). Immunofluorescence staining for F4/80 (right). Merged image. Bar, 100 μm. **C**: Quantitative analysis of F4/80-positive cells. Bars represent the average number of infiltrated macrophages in each CNV lesion. Values are mean±SEM (n=13 to 15); *n* represents the number of CNV lesions. *, p<0.05.

Furthermore, real-time PCR showed that mRNA expression of *F4/80*, the marker for mouse macrophages [18], was downregulated by 41% in the animals treated with 20 pmol SRPIN340 (n=6; n represents the number of eyes) compared to that of the vehicle-treated animals (n=8, p<0.05; Figure 5A). In accord with the real-time PCR data, suppression of macrophage influx by SRPIN340 was also depicted in the immunofluorescence study using F4/80 antibody. Compared with the vehicle-treated animals, the number of F4/80-positive macrophages in CNV lesions significantly decreased in the animals treated with 20 pmol SRPIN340 (15.5±4.0 and 6.5±1.8 /1000 μm^2^, n=13 to 15, respectively; n represents the number of CNV lesions; p<0.05; Figure 5B,C).

## Discussion

To date, intravitreal injection of anti-VEGF agents has brought revolutionary changes to the treatment of neovascular AMD. Nevertheless, novel interventions for preventing and treating neovascular AMD remain to be developed because of possible ocular and/or systemic adverse events following long-term administration [19-23]. Discovery of antiangiogenic *VEGF* isoforms highlighted the importance and necessity of differentially targeting *VEGF* isoforms in treating neovascular AMD, and suggested the possibility of regulating the splicing of *VEGF* pre-mRNA as a therapeutic strategy. Small molecules and compounds, including SRPIN340, have currently shown promising results in targeting spicing factors and kinases involved in alternative splicing [14,24,25].

Most of the findings regarding the regulation of alternative splicing of *VEGF* pre-mRNA during pathological angiogenesis were obtained from in vitro study [13,15]. Using in vitro experiments, upregulation of proangiogenic *VEGF* isoforms and simultaneous downregulation of antiangiogenic *VEGF* isoforms in various pathologies have subsequently been elucidated [26-29]. In contrast, animal experiments used in investigations of this mechanism are rarely reported. Moreover, a limited number of reports are currently available on the effect of specific inhibitors for SRPK against pathological angiogenesis such as a murine hypoxia-induced retinopathy model [15] and xenotransplanted tumor growth in nude mice [30]. Previous reports suggested that this suppressive effect was caused by the upregulation of antiangiogenic *VEGF* isoforms and the subsequent restoration of the balance between pro- and antiangiogenic *VEGF* isoforms. However, our data indicated that SRPIN340 suppressed total *Vegf* and *Vegf* isoforms containing exon 8a at the mRNA level. In addition, the total VEGF protein was also decreased by the SRPK inhibitor SRPIN340. Recently, Tripathi et al. reported that SRSF1 is a component of the 7SK complex and influences RNA polymerase II-mediated transcription [31]. Since SRPK activates SRSF1 through the phosphorylation of the RS domain [32], the current data suggest that SRPK inhibition by SRPIN340 may reduce transcription of *VEGF*, in addition to the switching to antiangiogenic *VEGF* isoforms.

During CNV formation, VEGF and its related molecules reciprocally accelerate angiogenesis via macrophage infiltration. For instance, VEGF induces MCP-1 and ICAM-1 production in endothelial cells, both of which play a role in macrophage recruitment [33-35], and directly mediates macrophage migration via its receptor 1 [36]. Subsequently, the recruited macrophages secrete VEGF and facilitate CNV growth [37]. Thus, decrease of VEGF might result in reduced macrophages influx and VEGF secretion. Indeed, in the current study we observed that levels of inflammation-associated molecules were decreased during CNV formation, following VEGF downregulation via SRPK inhibition with SRPIN340. Therefore, SRPK inhibition with SRPIN340 could disrupt the vicious cycle formed by VEGF and its related proteins such as MCP-1 and ICAM-1, and result in the attenuation of CNV formation, suggesting the possibility of a novel chemical treatment strategy for pathological angiogenesis, which targets SRPK.
